# Administration of zinc against arsenic-induced nephrotoxicity during gestation and lactation in rat model

**DOI:** 10.15171/jnp.2017.13

**Published:** 2016-12-25

**Authors:** Davood Nasiry Zarrin Ghabaee, Fereshteh Talebpour Amiri, Amir Esmaeelnejad Moghaddam, Ali Reza Khalatbary, Mehryar Zargari

**Affiliations:** ^1^Department of Anatomy, Student Research Committee, Mazandaran University of Medical Sciences, Sari, Iran; ^2^Department of Anatomy, Molecular and Cell Biology Research Center, Mazandaran University of Medical Sciences, Sari, Iran; ^3^Department of Clinical Biochemistry,​ Molecular and Cell Biology Research Center, Faculty of Medicine, Mazandaran University of Medical Sciences, Sari, Ira

**Keywords:** Arsenic, Zinc, Nephrotoxicity, Gestation, Lactation

## Abstract

**Background:**

Free radicals production by toxicity of arsenic (Ar) is most important in the nephrotoxicity. There is accumulating evidence that zinc (Zn), has anti-oxidant properties.

**Objectives:**

The aim of present study was to evaluate protective and ameliorative effects of Zn against Ar-induced nephrotoxicity in rat pups during gestation and lactation.

**Materials and Methods:**

Twenty-four adult pregnant wistar rats were randomly divided into four groups (n = 6). Group one was given vehicle only. Group two received Zn (ZnSO4) at 20 mg/kg/d. Group three received Ar at 5 mg/kg/d as sodium meta-arsenite. Group four received Ar + Zn at the same dose that mentioned in groups of two and three. At the end of the study, 24 hours after the last treatment, samples were killed with overdose of sodium pentobarbital and kidneys were harvested for measuring malondialdehyde (MDA), glutathione (GSH) and histopathological assessment.

**Results:**

The MDA level in kidney was increased in the Ar group, which was decreased after Zn administration in the Ar + Zn group. The GSH level in kidney was decreased in the Ar group, which were increased after Zn administration in the Ar + Zn group. Also, the histopathological changes which were detected in the Ar group attenuated after Zn consumption.

**Conclusions:**

Our findings suggested that administration of Zn during gestation and lactation could have protective and prevent effect in Ar-induced oxidative stress in kidney tissue.

**Implication for health policy/practice/research/medical education:**

In this experimental study, we found that Zn as an antioxidant agent and ability to cross of placenta, can protect kidney against Ar induced nephrotoxicity during gestation and lactation. The main mechanism of Zn in renoprotective effects was inhibition of oxidative stress by amelioration of lipid peroxidation produced as well as elevation of GSH.

## 1. Background

Arsenic (Ar) is a natural component of the earth’s crust and is widely distributed throughout the environment in the air, water, and land (1). It is severely toxicant in its inorganic form (2). The largest threat from Ar originates to public health is contaminated groundwater. Food prepared with contaminated water, drinking-water and crops irrigated with contaminated water are the sources of exposure (3). Chronic Ar exposure has been related to cancers of the skin, bladder, lungs and possibly the liver, as well as to dysfunction of renal and nervous systems (4). Also, long-term effects of Ar exposure during gestation can lead to preterm birth, low birth weight and resulting in high mortality and morbidity (5). Moreover, if mother exposed to higher levels of Ar, it may cause problems

like miscarriage and birth defects (6). The spectrum of defects includes exencephaly, encephaloceles, skeletal defects, and anomalies of the urogenital system (7). They can cause problems in overall health, in how the body develops, or in how the body works (8). In this regard, Ferm showed that exposure with high levels (30 mg/kg or more) of Ar would kill all of the embryos in utero, but low dosage levels (5-10 mg/kg) induced teratogenic effects (7) that one of the most important effects was renal agenesis (9). In another study, it was founded that Ar trioxide poisoning in pregnancy, crosses through the placenta at term with extremely high levels in fetal kidneys (10). Although the placenta is a barrier which prevents the transfer of some toxic metals to the fetus (Cd), but, it has not completely able to protective effect on the fetus against exposure to others toxics (Pb, Hg and Ar) (11). Its readily crosses the placental barrier and thus affects fetal development. Numerus investigation had detected, reproductive and developmental effects of Ar on humans and animal organs have been reported (9). Recently, it was reported that exposure to Ar in pregnancy period, can create renal agenesis, and cause degenerative changes in kidney tissue of fetus (12-14). The first pathogenic mechanism of Ar is production of oxidative stress due to antioxidant defense system dysfunction by increasing free radicals (15,16). Thus, it seems that the use of antioxidant supplemental with the cross of placental barrier ability can prevent side effect of Ar in pregnancy.

Zinc is the main source of antioxidants. Two mechanisms have been mentioned for that; first, the protection of sulfhydryl groups against oxidation and second, the inhibition of the production of reactive oxygen’s (17) by transition metals (18). There is accumulating evidence that attributed the beneficial effects of zinc to an anti-teratogenic, so that, in the pregnancy period, zinc deficiency can be considered as a teratogen for central nervous system and fetal skeletal (19). The other advantages of zinc are variety of biological activities (20). So that, recent studies documented that following co-administration of zinc, resulted in significantly increased in the levels of glutathione (GSH) and glutathione-S-transferase (GST) (21), as well as a significant decrease in the levels of superoxide dismutase (SOD) and lipid peroxidase (MDA) (22). Also, zinc plays important roles during embryogenesis, fetal growth, and milk secretion (23). However, the effects of this chemical on Ar-in­duced nephrotoxicity during the gestation and lactation has not been reported.

## 2. Objectives

The purpose of the present study is to investigate the protective effects of zinc consumption against Ar induced-nephrotoxicity in offspring during the gestation and lactation.

## 3. Materials and Methods

### 3.1. Animals

Adult female Wistar rats (250-300 g) were used from laboratory animal research center, Sari, Iran in this study. They were kept in the laboratory under constant conditions of temperature (23±2ºC) and in a reverse 12 hours dark/light cycle (lights on at 20:00 pm) for at least 1 week before and through the experimental work. After this period, they were mated with males. A sperm-positive vaginal smear test was taken to detect the first day of pregnancy. From this time, rats were housed individually in cages and maintained under the supervision according to the guidelines of the university’s animal care codes.

### 3.2.Study design and treatment

The animals were randomly allocated to four groups, each containing 6 rats: (І) Control group, which received distilled water; (ІІ) Zinc (Zn) treated group, which received Zn (ZnSO4) at 20 mg/kg/d prepared in distilled water (24,25); (ІІІ) Ar treated group, which received Ar at 5 mg/kg/d as sodium meta-arsenite (Merck^®^) prepared in distilled water (26); (ІV) Ar + Zn treated group which received Ar and Zn at the same dose that mentioned in groups of two and three. All groups were treated via gavaging during gestation and lactation. Twenty-four hours after the last treatment (experimental period: 42 days: 21 of gestation and 21 of lactation), 2-4 pups were culled at randomly from each rat in each group. Then, all pups were killed with overdose of sodium pentobarbital and kidneys were harvested for biochemical and histopathological assessments.

### 3.3. Biochemistry

The collected samples (1 kidney from each pups) were abstergent by PBS in order to thoroughly cleaned of blood, then were immediately frozen and stored in a -80°C freezer for biochemical analysis. Two enzymes were assessed. First, malondialdehyde (MDA) (27) and second, GSH levels (28), employing flame atomic absorption spectrometry according to standard methods (17).

### 3.4. Histopathological studies

The obtained samples were thoroughly cleaned of blood, and then were immediately fixed in 10% (w/v) buffered formalin for 24 hours, embedded in paraffin. Five-micrometer serial sections were prepared from the paraffin-embedded blocks using by microtome. For histopathological assessment, some tissue sections were deparaffinized with xylene, stained with hematoxylin and eosin (H&E), and also periodic acid–Schiff (PAS) and studied by light microscopy (DME; Leica Microsystems Inc., Buffalo, NY, USA) to assess the histopathological changes.

Tubular damage (in 5 fields/each section) was scored by using the percentage of cortical tubules that showing epithelial necrosis: 0 index; <25% damage was assigned 1; 25%-50% damage was assigned 2; 50%-75% damage was assigned 3 and >75% damage was assigned a 4 index. The means of tubular necrosis was the blebbing of apical membranes, loss of the proximal tubular brush border, tubular epithelial cell detachment from the basement membrane or intraluminal aggregation of cells and proteins (29,30). All the histological studies were performed in a blinded fashion.

### 3.5. Ethical issues

The research followed the tenets of the Declaration of Helsinki. The research was approved by ethical committee of Mazandaran University of Medical Sciences. Prior to the experiment, the protocols were confirmed to be in accordance with the guidelines of Animal Ethics Committee of Mazandaran University of Medical Sciences (Ethical code; IR.MAZUMS.REC.95.S203).

### 3.6. Statistical analysis

Statistical analysis was carried out in SPSS (version 15, Chicago, IL, USA). Results were presented as mean values (±SD). The Kolmogorov–Smirnov test was used in order to evaluate the normality of the data. The Tukey‏׳s multiple comparison tests and the analysis of the variance were used to compare each two groups and data among the groups, respectively. A value of P < 0.05 was considered significant.

## 4. Results

### 4.1. General health

In comparison with control, Zn and Ar + Zn groups, influence of Ar treatment on body or organ weights was significant (*P* < 0.05). Also, in comparison with control and Zn groups, total birth numbers and infants dead in Ar group had significant decreased and increased, respectively (*P* < 0.05; Table 1).

**Table 1:** Effect of zinc against arsenic-induced nephrotoxicity in rat pups during gestation and lactation

	Control	Zn	Ar	Ar+Zn
Infant total weight (g)	45.2 ± 4.6	46.4 ± 6.65	37.2 ± 1.3^*^	42.2 ± 2.38
Tissue weight (mg)	71.6 ± 9.07	64 ± 6.92	45.68 ± 5.85^*^	57 ± 2.64
Total birth numbers	9.33 ± 1.96	8 ± 2.66	4.66 ± 2.06^*^	7.16 ± 1.83
Infant dead	0.0 ± 0.0	0.0 ± 0.0	1.16 ± 1.1^*^	0.16 ± 0.1

*P < 0.05 versus control, Zn and Ar + Zn groups. All values are expressed in Mean ± SD.

### 4.2. General health

**Figure 1:**
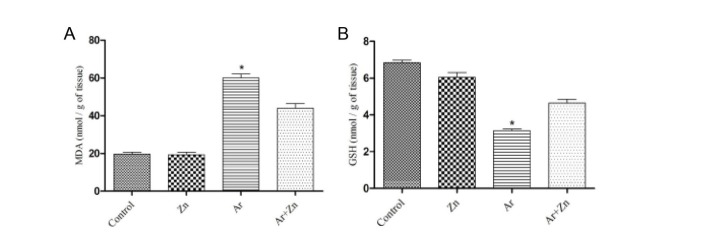
(A) Effect of zinc against arsenic-induced nephrotoxicity in rat pups during gestation and lactation. (B) Effect of zinc against arsenic-induced nephrotoxicity in rat pups during gestation and lactation. All values are expressed in Mean ± SD (n = 18–24). *P *lt; 0.001 versus other groups.

MDA levels in the Ar group was a significant elevation (*P* < 0.05) compared to other groups. The MDA levels in the Ar + Zn group were significantly lower than those of the Ar group (*P* < 0.05; Figure 1A). Also, about GSH levels, the results showed that administration of Ar in the Ar group produced a significant decrease (*P* < 0.05) in GSH level compared to the other groups. We found the GSH levels significantly increased (*P* < 0.05) in Ar+Zn group compared to Ar group (Figure 1B).

### 4.2. Histopathological Changes

**Figure 2:**
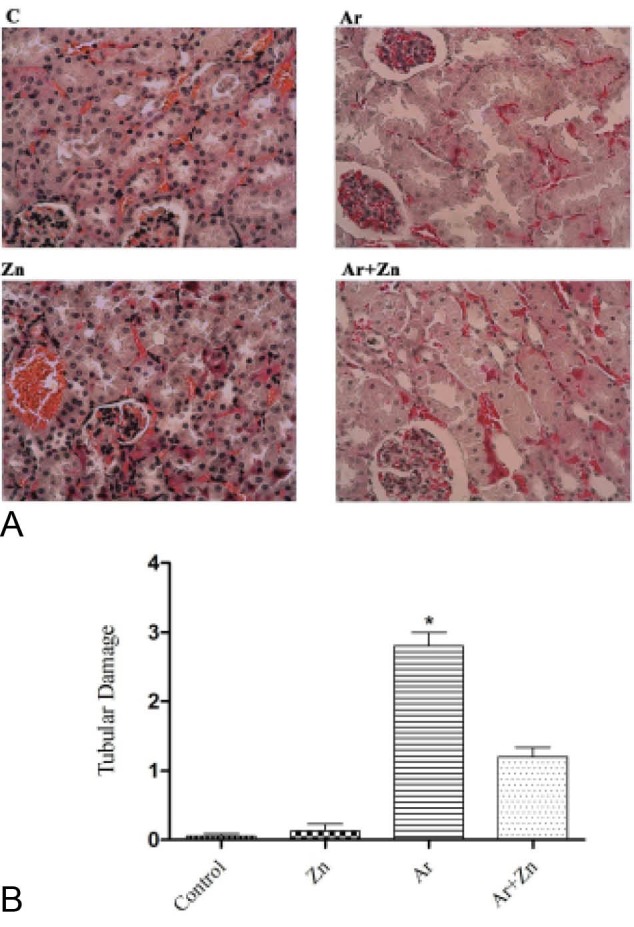
Effect of zinc against arsenic-induced nephrotoxicity in rat pups during gestation and lactation. Microscopic examination of sections stained with PAS at ×400 magnification. Animals were divided into four groups: the control group (C), the zinc groups (Zn) (25), the arsenic group (Ar) and the arsenic + zinc group (Ar+Zn). (A) The structure of the kidney was normal in the C and Zn groups. In the Ar group, the renal veins were enlarged and congested with blood, the renal tubules showed wide lumen, and the glomeruli were atrophied. Also, cystic dilatations of the bowman capsule, renal tubular epithelial cell swelling and pyknotic nuclei of renal epithelium were observed. Compared to the Ar group, the Ar+Zn group showed a little structural damage. (B) Tubular necrosis was scored in harvested renal tissues tubular damage. Data are presented as Mean ± SD. *P<0.001 versus other groups.

Results of histopathological examination following PAS staining are shown in Figure 2A. In Ar group, we observed renal tubular epithelial cell swelling, brush border damage, degeneration, necrosis, tubular casts, cell vacuole degeneration in the proximal tubules, pyknotic nuclei of renal epithelium and the glomeruli were atrophied, while in the control and Zn groups, the kidneys maintained normal structure. Compared to the Ar group, treatment with Zinc in Ar + Zn animals ameliorated these histopathological alternations, so that only a little nuclear pyknosis of renal epithelium and mild dilation of the bowman capsule and renal tubules were observed. We further calculated scores of tubular damage as shown in Figure 2B. The Ar group had an injury score of 3, while the Ar + Zn group scored 1.25, and this difference was significant (*P* <0.001).

## 5. Discussion

The main findings of the current study showed that administration of zinc during gestation and lactation to mother, can attenuate histopathological changes, inflammation, and lipid peroxidation in pups. Meanwhile, it improves antioxidant capacity in kidney tissue against Ar-induced nephrotoxicity.

Kidney with excretion of methylated species of Ar is considered as a target organ in Ar toxicity (31). So, chronic exposures to Ar have some of undesirable effects on different organs, including the kidney. In this regard, studies have shown that Ar administration increased significantly kidney agenesis and can be killer, as an indicator of lipid peroxidation, in rats compared with the control group (32,33). However, consequences of Ar exposure and protective effect of zinc during gestation and lactation on kidney tissue are still poorly understood. Therefore, present experiment is a first study to investigate the protective effect of zinc against nephrotoxicity caused by Ar during gestation and lactation in rat model.

Endogenous antioxidants prevent cellular oxidative damage caused by free radicals. Studies have shown that the total antioxidant capacity and GSH levels in kidney tissues were significantly decreased after Ar administration compared to control groups (34). Also, lipid peroxidation is an important pathologic event, polyunsaturated fatty acids’ breakdown, which is induced by free radicals (35).

Our results showed that exposure of Ar during gestation and lactation increased MDA and decreased GSH levels whereas administration of zinc attenuated in the treatment group with Ar. Ar due to binds to the glomerular membrane, it can be lipophilicity and increases lipid peroxidation (32). Also, previous reports documented that MDA production in Ar-exposed was increased (29,36), this indicates the main mechanism of Ar toxicity caused by oxidative stress (37). In this study, Ar significantly increases MDA production, so this finding is agreed with other study and with the same dose (36). Another anzyme assessed in present study was GSH. GSH by having a sulfhydryl (31) group acts as a scavenger of free radicals and reduces lipid peroxidation (38). Ar by connecting to sulphydryl group-containing compounds inhibits the GSH reductase and produces excessive ROS in kidney which this factor can conduce the organ damage (33). Our results showed that administration of Ar decreased GSH levels in the kidney tissues, meanwhile the decrease somewhat attenuated after administration of zinc in the treatment group. In this regard, it was documented that zinc restored the antioxidant status in kidney after cyclosporine- and mercuric chloride-induced nephrotoxicity in rats.

Zinc has antioxidant properties and by absorbing free radicals, reduces lipid peroxidation level can prevention of oxidative stress (34). On the other hand, the interaction between zinc and other metals in the development of mammals would be antagonistic action; When zinc in the diet increased more than the required level, reduces the absorption of trace metals (39).

Although the majority of zinc absorption is through the mouth and proximal intestine, studies revealed that placental zinc transport is by microvillus border membranes and cultured syncytiotrophoblasts (23). Therefore, it can be concluded zinc consumption leading to increases total plasma antioxidant activity.

Furthermore, the quality of the maternal diet has a dominant effect on embryonic and fetal development and a significant moderating effect has on the expression of developmental toxicity of some agents (40).

In this study, difference was observed in total weight in rat pups that exposed to Ar; this result was in contrast with Pineda et al studies (36), who reported that the administration of Ar in drinking water did not alter this parameter. In this regard, in present study Ar was received by gavage. On the other hand, pups treated with zinc had more weight and total birth numbers and infants dead had significant decreased than Ar group. So this confirms that the zinc is important in the development pre- and postnatal (41). In fact, the use of zinc supplements increases birth weight and decreases related pregnancy complications (42).

Histopathological findings showed less damage in the group that received zinc concurrent with Ar in compared to Ar group. The reduced degree of pyknosis of renal epithelium and mild dilation of the bowman capsule and renal tubules in Ar plus zinc group again implied the nephroprotective effect of zinc, but compared with Ar group was not significant. Perhaps it could be due to lack of evolution is completely all at birth. New nephrons in the kidney cortex margin, their maturity gained in a few days after birth. At this time, the amount of Ar that passes through breast milk is relatively insignificant (43). Zinc administration significantly effected on the progression of renal histopathological changes, which represents the antioxidant potential of Zinc.

## 6. Conclusions

Our results support that zinc – a good source of antioxidant – can markedly attenuate the indicators of the Ar-induced nephrotoxicity during gestation and lactation; so it can be recommended as a dietary supplement to reduce the side effects of synthetic pyrethroid in during pregnancy.

## Authors’ contribution

DN and FT; study design, data gathering and data interpretation, preparation of manuscript and final revision. ARK and AE; consultant of study. MZ; performed the biochemical tests. All authors read and approved the paper.

## Conflicts of interest

None declared

## Funding/Support

This study was supported by Student Research Committee and Molecular and Cell Biology Research Center of Mazandaran University of Medical sciences (Grant No: 95/21).
